# Caffeine Protects Against Retinal Inflammation

**DOI:** 10.3389/fphar.2021.824885

**Published:** 2022-01-06

**Authors:** Federica Conti, Francesca Lazzara, Giovanni Luca Romano, Chiara Bianca Maria Platania, Filippo Drago, Claudio Bucolo

**Affiliations:** ^1^ Department of Biomedical and Biotechnological Sciences, School of Medicine, University of Catania, Catania, Italy; ^2^ Center for Research in Ocular Pharmacology-CERFO, University of Catania, Catania, Italy

**Keywords:** caffeine, inflammation, retina, BDNF, retinal pigment epithelial cells

## Abstract

Caffeine, one of the most consumed central nervous system (CNS) stimulants, is an antagonist of A_1_ and A_2A_ adenosine receptors. In this study, we investigated the potential protective effects of this methylxanthine in the retinal tissue. We tested caffeine by using *in vitro* and *in vivo* paradigms of retinal inflammation. Human retinal pigment epithelial cells (ARPE-19) were exposed to lipopolysaccharide (LPS) with or without caffeine. This latter was able to reduce the inflammatory response in ARPE-19 cells exposed to LPS, attenuating the release of IL-1β, IL-6, and TNF-α and the nuclear translocation of p-NFκB. Additionally, caffeine treatment restored the integrity of the ARPE-19 monolayer assessed by transepithelial electrical resistance (TEER) and the sodium fluorescein permeability test. Finally, the ischemia reperfusion (I/R) injury model was used in C57BL/6J mice to induce retinal inflammation and investigate the effects of caffeine treatment. Mouse eyes were treated topically with caffeine, and a pattern electroretinogram (PERG) was used to assess the retinal ganglion cell (RGC) function; furthermore, we evaluated the levels of IL-6 and BDNF in the retina. Retinal BDNF dropped significantly (*p* < 0.05) in the I/R group compared to the control group (normal mice); on the contrary, caffeine treatment maintained physiological levels of BDNF in the retina of I/R eyes. Caffeine was also able to reduce IL-6 mRNA levels in the retina of I/R eyes. In conclusion, these findings suggest that caffeine is a good candidate to counteract inflammation in retinal diseases.

## Introduction

Caffeine is the 1,3,7 trimethylxanthine and represents one of the most consumed central nervous system (CNS) stimulants, with an average assumption within 100–400 mg per day (Sc and Muralidhara, 2016), through consumption of coffee, tea, and soft drinks enriched with caffeine (Mitchell et al., 2014), along with caffeine supplements, generally used as metabolism boosters (Gurley et al., 2015). Caffeine is endowed with anti-inflammatory and antioxidant properties, as reported by several studies on different models of neurodegenerative diseases, including Parkinson’s disease and Alzheimer’s disease (Dall’lgna et al., 2003; Carman et al., 2014; Xu et al., 2016; Hosny et al., 2019). Moreover, caffeine has shown beneficial effects in several pathologies, such as chronic stress, diabetes, attention deficit, and hyperactivity disorders (Park et al., 2007; Alves et al., 2020; Ibrahim et al., 2020). Caffeine is a non-selective adenosine receptor (AR) antagonist, although it has a higher affinity for the adenosine A_1_ receptor (A_1_R) and the adenosine A_2A_ receptor (A_2A_R); furthermore, caffeine is a non-selective inhibitor of phosphodiesterases (PDEs) (Jacobson et al., 2020). A_1_R and A_2A_R are G-protein–coupled receptors (GPCRs), and they are expressed in human retinal pigment epithelial (RPE) cells (Wan et al., 2011) and in other layers of the retina (Fredholm et al., 2011; Wurm et al., 2011; Liu et al., 2018).

Caffeine has provided neuroprotective action through the blockade of A_2A_R (Xu et al., 2016; Boia et al., 2017). In consideration of the complex pharmacological profile of this drug, the effects of caffeine are not straightforward to be predicted (Dai and Zhou, 2011), and although the current literature provides some evidence regarding the effects of caffeine in the CNS, few studies were carried out regarding its actions in the eye. Considering the similarities between neurodegenerative diseases of the brain and retina (Romano et al., 2015, Romano et al., 2017; Platania et al., 2017), we investigated caffeine by using *in vitro* and *in vivo* paradigms of retinal inflammation. The retinal inflammatory process occurs in several ocular diseases such as age-related macular degeneration (AMD) and diabetic retinopathy (DR). This latter is one of the leading causes of irreversible vision loss in industrialized countries and represents a severe retinal degenerative disease (Van Lookeren Campagne et al., 2014). AMD is mainly characterized by accumulation of the pigment lipofuscin in the RPE cells (Katz, 2002) and by retinal ischemia (Rivera et al., 2017). About 15% of AMD patients switch to the wet form, characterized by choroidal neovascularization. Currently, approved pharmacological treatments such as anti-VEGF agents and steroids are available only for the wet form of AMD and DR (Bucolo et al., 2005; Sarao et al., 2014; Giurdanella et al., 2015; Amadio et al., 2016; Campochiaro et al., 2016; Bucolo et al., 2018); no treatments have been approved yet for the dry form of the disease where the inflammation is prevalent, and it is considered a hallmark of the early phase of this condition. Protection of RPE cells and retinal ganglion cells (RGCs), along with blood retinal barrier (BRB) preservation, can be considered as a new strategy to prevent the devastating damage of retinal degenerative diseases. It has been widely demonstrated that the activation of toll-like receptor 4 (TLR-4), induced by LPS, stimulates the nuclear translocation of the nuclear factor kappa-light-chain-enhancer of activated B cells (NF-κB) and, as a direct consequence, the over-expression of inflammatory cytokines, such as interleukin-6 (IL-6), interleukin-1β (IL-1β), or tumor necrosis factor-alpha (TNF-α) (Holtkamp et al., 1998; Izumi-Nagai et al., 2007; Salminen and Kaarniranta, 2009; Do et al., 2020). It is noteworthy that AMD patients have increased vitreous levels of IL-1β (Tang and Kern, 2011; Zhao et al., 2015; Dabouz et al., 2020) and plasmatic tumor necrosis receptor 2 (TNF-R2) (Krogh Nielsen et al., 2019). Usually, RPE cells release neurotrophic factors such as the brain-derived growth factor (BDNF), which is a key factor for survival and function of RGCs and photoreceptors (Ponnalagu et al., 2017; Bahrami et al., 2019). Furthermore, low BDNF levels have been found in the aqueous humor of AMD patients, causing an insufficient protection of the retinal tissue (Inanc Tekin et al., 2018). The aim of the present study was to explore the neuroprotective and the anti-inflammatory effects of caffeine in two models of retinal inflammation by using human RPE cells and C57BL/6J mice, respectively.

## Materials and Methods

### Cell Culture

Human retinal pigment epithelial cells (ARPE-19) were purchased from ATCC^®^ (Manassas, Virginia, United States). Cells were cultured at 37°C (humidified atmosphere with 5% CO_2_) in ATCC-formulated DMEM:F12 medium (ATCC number 30–2006, Manassas, Virginia, United States) with 100 U/mL penicillin, 100 μg/ml streptomycin, and 10% fetal bovine serum (FBS). After reaching confluence (∼70%), ARPE-19 were pretreated for 24 h with caffeine at concentrations of 1, 10, 100 and 1000 µM (Sigma-Aldrich, Cat.No. C0750, St Louis, MO) and/or 1 µM of CGS 21680 hydrochloride (Tocris Bioscience, Cat.No. 1063, Bristol, United Kingdom) (Wang et al., 2014) in DMEM:F12 supplemented with only 5% FBS to starve cells. In control cells (untreated), only fresh medium has been added. After pretreatment, ARPE-19 were challenged with 150 ng/ml, 2 μg/ml, or 10 μg/ml of lipopolysaccharide *E. coli* (LPS) (Enzo Life Sciences ALX-581–010-L001, Farmingdale, NY) to simulate inflammation and also with different concentrations of caffeine (1–100 µM) and/or 1 µM of CGS 21680.

### MTT Assay

The 3-[4,5-dimethylthiazol-2-yl]-2,5-diphenyl tetrasodium bromide (MTT; Chemicon, Temecula, CA) was used to assess cell viability. Optimal cell density was obtained by seeding 3 × 10^4^ cells/well in 96-well plates (Costar, Corning, New York). After 24 h of culture, ARPE-19 were treated with caffeine (1–1000 µM), in medium containing FBS 10% for 48 h. At the end of the treatment, ARPE-19 were incubated at 37°C with MTT (0.5 mg/ml) for 2 h; then, DMSO 100 µL per well was added, and absorbance was measured at 570 nm in a plate reader (VariosKan, Thermo Fisher Scientific, Waltham, MA). Results were reported as the percentage of control.

### LDH Assay

Lactate dehydrogenase (LDH) cell release was measured using the Cytotoxicity Detection KitPLUS (LDH) (Roche Diagnostics 04744934001, Basel, Switzerland). ARPE-19 cells were seeded at 3 × 10^4^ cells/well in 96-well plates (Costar, Corning, New York). After reaching confluence, cells were treated for 48 h with caffeine (1–1000 µM), in medium containing FBS 10%. After treatment, according to the manufacturer’s protocol, lysis solution was added to positive control wells (non-treated cells) for 15 min. After transferring 100 µL of the medium in a new multi-well, 100 µL of the working solution was added. After 10–15 min at room temperature, 50 µL of the stop solution was added lastly. The absorbance values were measured at 490 nm using a plate reader (VarioSkan, Thermo Fisher Scientific, Waltham, MA). LDH release is reported as LDH (% control): (abs_x_ ÷ abs_ctrl+_) × 100. In the equation, abs_x_ is absorbance in the x well, and abs_ctrl+_ is the average absorbance of positive control cells (untreated lysed cells). Absorbance values were edited by removing the blank.

### Extraction of Total RNA and cDNA Synthesis

Extraction of total RNA, from ARPE-19 and mouse retinas, was performed with TRIzol Reagent (Invitrogen, Life Technologies, Carlsbad, CA, United States). The A_260_/A_280_ ratio of the optical density of RNA samples (measured with Multimode Reader Flash di Varioskan™) was 1.95–2.01; this RNA purity was confirmed by electrophoresis in the non-denaturing 1% agarose gel (in TAE). cDNA was synthesized from 2 µg (ARPE-19) and 500 ng (mice retinas) of RNA with a reverse transcription kit (SuperScript™ II Reverse transcriptase, Invitrogen, Thermo Fisher Scientific, Carlsbad, CA, United States).

### Real-Time Reverse Transcriptase-Polymerase Chain Reaction (RT-PCR)

Real-time RT-PCR was carried out with the Rotor-Gene Q (Qiagen, Germantown, MD, United States). The amplification reaction mix included the Master Mix Qiagen (10 µL) (Qiagen QuantiNova SYBR Green Real-Time PCR Kit, Germantown, MD, United States) and cDNA (1 µL,100 ng). Forty-five amplification cycles were carried out for each sample. Results were analyzed with the 2^−ΔΔCt^ method (Leggio et al., 2019). Quantitative PCR experiments followed the MIQE guidelines. Gene expression levels were normalized with levels of housekeeping gene (18S). Primers were purchased from Eurofins Genomics (Milan, Italy) and Qiagen (Milan, Italy). Forward and reverse primer sequences (for human and mouse genes) and catalogue numbers are herein listed: human IL-1β (Forward: 5′-AGC​TAC​GAA​TCT​CCG​ACC​AC-3'; Reverse: 5′-CGT​TAT​CCC​ATG​TGT​CGA​AGA​A-3′), human IL-6 (Catalogue Number QT00083720), human TNF-α (Forward 5′-AGC​CCA​TGT​TGT​AGC​AAA​CC-3'; Reverse 5′-TGA​GGT​ACA​GGC​CCT​CTG​AT-3′), human 18S (Forward 5′-AGT​CCC​TGC​CCT​TTG-3'; Reverse 5′-GAT​CCG​AGG​GCC​TCA​CTA​AAC-3′), human BDNF (Catalogue Number QT00235368), mice 18S (Forward: 5′-GTT​CCG​ACC​ATA​AAC​GAT​GCC-3′; Reverse: 5′-TGG​TGG​TGC​CCT​TCC​GTC​AAT-3′), mice BDNF (Forward: 5′-GTT​CGA​GAG​GTC​TGA​CGA​CG-3′; Reverse: 5′-AGT​CCG​CGT​CCT​TAT​GGT​TT-3′), and mice IL-6 (Cat. No. QT00098875).

### Western Blot

ARPE-19 were cultured in 60 mm petri dishes at a density of 1,3 × 10^6^. After 24 h of pretreatment with caffeine (1–100 µM) and/or CGS 21680 (1 µM) and co-treatment with 10 μg/ml of LPS for 2 h, cytoplasmic and nuclear proteins were extracted by using the CER/NER kit (NE-PER Nuclear and Cytoplasmic extraction reagents,78,833, Invitrogen, Life Technologies, Carlsbad, United States) according to the manufacturer’s protocol. The protein content was determined by using the BCA Assay Kit (Pierce™ BCA Protein Assay Kit, Invitrogen, Life Technologies, Carlsbad, United States). Extracted proteins (20 μg) were loaded on the NuPAGE ^TM^ 10% Bis-Tris mini protein gel (Invitrogen, Life Technologies, Carlsbad, CA, United States). After electrophoresis, proteins were transferred into a nitrocellulose membrane (Invitrogen, Life Technologies, Carlsbad, CA). Membranes were blocked with milk 5% in Tris-buffered saline 0.2% Tween 20 (TBST) for 1 h at room temperature. Membranes were incubated overnight (4°C) with appropriate primary phospho-NFκB p65 (Ser536; mouse mAb #3036 Cell Signaling Technology, MA, United States, 1:500 dilution), anti-β-Actin (Rabbit mAb #A2066 Sigma-Aldrich, St Louis, MO; 1:1000 dilution), and anti-lamin B (Mouse monoclonal IgG_2b_, sc-365214 Santa Cruz Biotechnology, INC, CA, United States; 1:1000 dilution) antibodies. After overnight incubation, the membranes were then incubated with secondary chemiluminescent antibodies (ECL anti-mouse, NA931 and ECL anti-rabbit, NA934, 1:2000 dilution) for 1 h at room temperature. After secondary antibodies, membranes were incubated with ECL (SuperSignal™ West Pico PLUS Chemiluminescent Substrate, 34,577, Thermo Fisher Scientific, Carlsbad, CA, United States) and were detected through I-Bright^TM^ 1500 (A43679, Invitrogen, Life Technologies, Carlsbad, CA, United States) by chemiluminescence. Densitometry analyses of blots were performed at non-saturating exposures and analyzed by ImageJ software (NIH, Bethesda, MD). The values were normalized to *β*-actin and lamin B, which were used as housekeeping control for cytoplasmic and nuclear fraction, respectively.

### Transepithelial Electrical Resistance (TEER) and Permeability Test

Transepithelial electrical resistance was measured by using a Millicell-Electrical Resistance System (ERS2) (Merck, Millipore, Burlington, MA, United States) as previously described (Giurdanella et al., 2017). TEER values were reported as ω×cm^2^ and were calculated as (average resistance of well–average resistance of the blank well) × 0.33 (the area of the membrane). ARPE-19 cells were seeded (1 × 10^5^ cells/well) in 24-well plates on cell culture transwell inserts (Falcon^TM^ 24 well 0.4 μm pore size, #353095, Becton Dickinson Labware, Bedford, MA, United States). After reaching confluence, cells were pretreated with caffeine (1–100 µM) for 24 h in DMEM:F12 supplemented with 5% FBS and next with LPS 2 μg/ml and caffeine (1–100 µM) for 24 h. To evaluate the BRB permeability, cell culture inserts were transferred in new 24-well plates, and a solution of sodium fluorescein (Na-F) (10 mg/ml) was added. After 5, 15, and 30 min, the quantification of fluorescence (Na-F: excitation 480 nm, emission 535 nm) was carried out using a Varioskan Flash microplate reader (Thermo Fisher Scientific, Waltham, MA, United States). Values were reported as previously described (Fresta et al., 2020).

### Immunocytochemistry

ARPE-19 cells were seeded at a density of 7 × 10^4^/well on 24-well glass chamber slides. After 3 days, cells were pretreated with caffeine (1–100 µM) for 24 h in DMEM F12 containing 5% of FBS. Subsequently, cells were subjected to LPS stimulus (10 μg/ml) for 72 h and different concentrations of caffeine. At the end of the treatment, cells were fixed with acetone for 15′ at -20°C and subsequently with methanol for 20′ at -20°C. After washing with PBS 1X, cells were permeabilized with Triton 0.2% for 5′ at 4°C. After permeabilization, cells were incubated with the primary antibody (Rabbit anti-ZO-1, 617300, Invitrogen, Life Technologies, Carlsbad, CA, United States; 1:100 in triton 0.1%) overnight at 4 °C. Then, cells were washed and incubated with the secondary antibody (Goat anti-rabbit, ab96899, Abcam, Cambridge, United Kingdom; 1:300 in triton 0.1%) for 1 h at room temperature in the dark. After washing again, the slides were mounted using Fluoroshield™ with DAPI (F6057-29ML Sigma-Aldrich, St Louis, MO). Images were acquired by using the Zeiss Observer Z1 microscope (Carl Zeiss Microscopy GmbH, Oberkochen, Germany). A semi-quantitative evaluation of the ZO-1 expression was carried out analyzing images from slides of each condition (*n* = 4) (Ctrl, LPS, LPS + caffeine 1 μM, LPS + caffeine 10 μM, LPS + caffeine 100 µM). The images (*n* = 4 per group) were analyzed, and ImageJ was used for measurements of the average gray scale.

### Animals

Male C57BL/6J mice (3 months of age) (Charles River Laboratories, Italy) were housed in a temperature-controlled environment with free access to food and water during a 12 h light–dark cycle. All animals were treated according to the Principles for the Care and Use of Animals in Ophthalmic and Vision Research approved by the Association for Research in Vision and Ophthalmology. University of Catania (Italy) Ethics Committee approval #343.

### Ischemic/Reperfusion Retina Damage

Retinal ischemia/reperfusion (I/R) has been used to induce retinal injury, as previously described in many rodent species (Osborne et al., 2004; Gustavsson et al., 2008; Ulbrich et al., 2017; Stankowska et al., 2019). Mice were anesthetized by tiletamine + zolazepam (60 mg/kg) and medetomidine (40 μg/kg) administered through intraperitoneal injection; moreover, 0.4% oxybuprocaine (Novesina^®^, Laboratoires Thea, Clermont-Ferrand, France) has been administered topically. The animals were placed on a heating pad to prevent hypothermia during the experiment. A 32-gauge needle, connected with a reservoir containing phosphate-buffered saline, was introduced into the anterior chamber through the cornea to increase the intraocular pressure (up to 90 mmHg). Retinal ischemia was confirmed by the observation of blanching of the anterior segment and arteries in the eye. After 60 min, the needle was removed to allow reperfusion. Ocular formulation of 1.9% caffeine was instilled (10 µL) 60 min before I/R and after reperfusion, twice a day for 72 h. Mice were euthanized after 72 h from I/R insult, the eyes were enucleated, and the retinas were collected to assess IL-6 and BDNF mRNA expressions.

### Pattern Electroretinogram (PERG)

PERG has been used as a sensitive measure of RGC function (Chou et al., 2018). Anesthetized mice were transferred on a heating plate with the mouse superior incisor teeth hooked to a bite bar and the head gently restrained by two ear knobs. The body was kept at a constant temperature of 37 °C using a feedback-controlled heating pad (TCAT-2LV, Physitemp Instruments, Inc, Clifton, NJ, United States). Two microliters of topical balanced salt solution (BSS) were applied to prevent corneal dryness. Simultaneous recordings of PERG response from both eyes were obtained using a common subcutaneous needle in the snout (jorvec Corp, Miami, FL, United States). To obtain PERG records, visual stimuli (black–white horizontal bars generated on LED tablets) are presented independently to each eye at 10 cm distance (56° vertical × 63° horizontal field; spatial frequency, 0.05 cycles/deg; 98% contrast; 800 cd/sqm mean luminance; left-eye reversal rate, 0.992 Hz; right-eye reversal rate, 0.984 Hz). Electrical signals recorded were averaged (>1,110 epochs), and PERG responses from each eye were isolated by averaging at stimulus-specific synchrony. PERG waveforms consist of a positive wave (defined as P1) followed by a slower negative wave with a broad trough (defined as N2). Therefore, each waveform has been analyzed by measuring the peak-to-trough (P1-N2) amplitude defined as the PERG amplitude and the time-to-peak of the P1 wave defined as PERG latency (Porciatti, 2014).

### Statistical Analysis

Statistical analysis was performed with GraphPad prism 7 (GraphPad software La Jolla, California). The data generated by all experiments are reported as mean ± SD (*n* = 4). One-way analysis of variance (ANOVA) was carried out, and Tukey’s *post hoc* test was used for multiple comparisons. Differences between groups were considered statistically significant for *p*-values < 0.05.

## Results

### Effects of Caffeine on ARPE-19 Cell Viability

**FIGURE 1:**
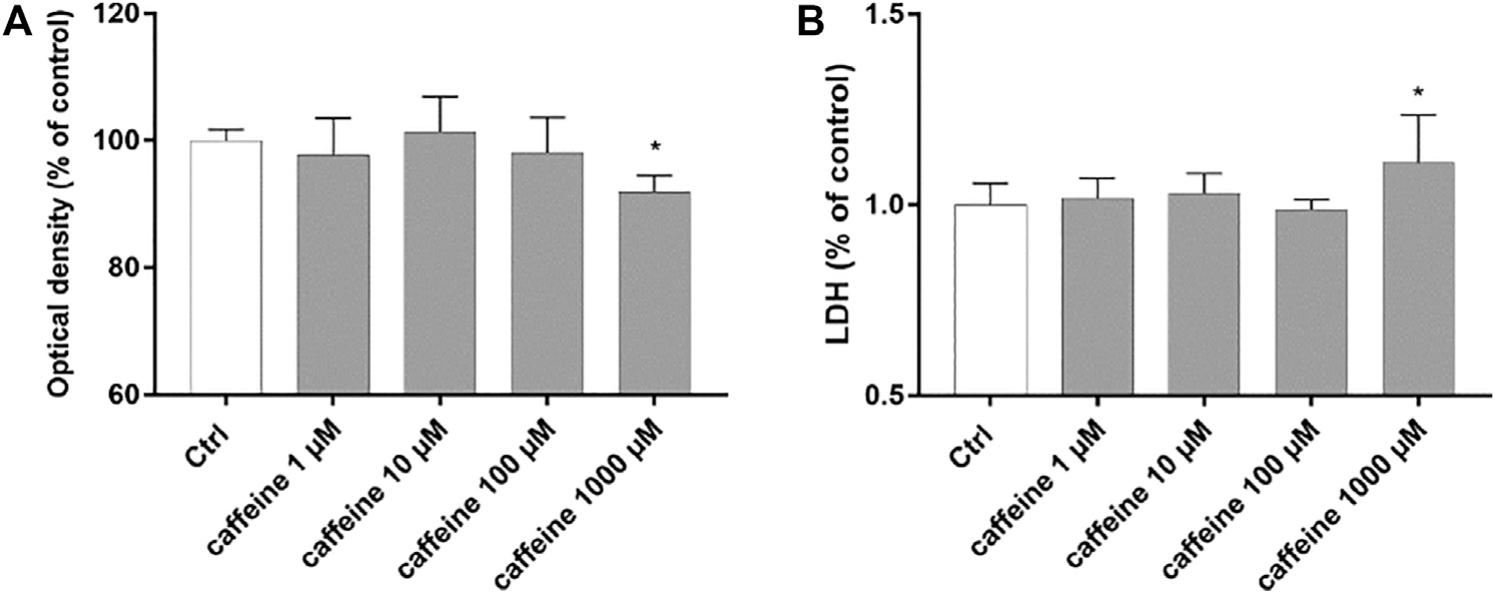
Caffeine 1–100 µM is tolerated by ARPE-19 cells. Caffeine (1–10–100 µM) after 48 h did not reduce cell viability **(A)** and did not increase LDH release **(B)**. Values are reported as mean ± SD; *n* = 4. Data were analyzed by one-way ANOVA and the Tukey *post hoc* test for multiple comparisons. *p < 0.05 *vs.* control.

Preliminary studies were carried out to evaluate cell viability and cytotoxicity after treatment with caffeine (1–1000 µM). At concentrations of 1–10 and 100 μM, caffeine did not reduce cell viability compared to control cells (Figure 1A). Moreover, as shown in Figure 1B, caffeine did not increase the LDH release, compared to untreated (control) cells, whereas treatment with caffeine 1000 µM led to a significant (*p* < 0.05) reduction of cell viability and to a significant (*p* < 0.05) increase of the LDH release (Figure 1A,B). For this reason, we excluded caffeine 1000 µM for all subsequent experiments.

### Effects of Caffeine on Inflammatory Markers and BDNF

**FIGURE 2:**
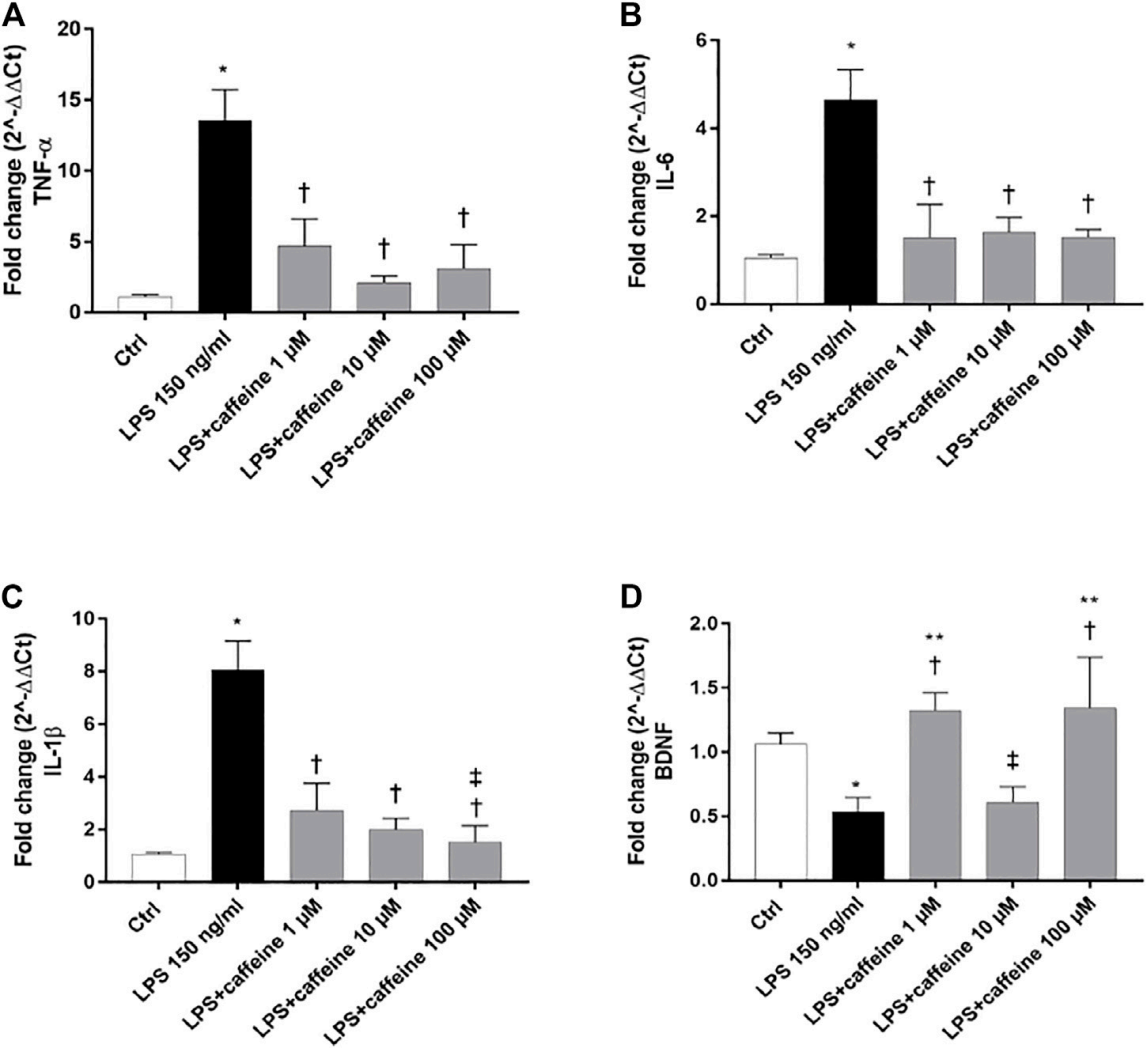
Caffeine counteracts inflammation and elicits the BDNF expression after LPS treatment. ARPE-19 were pretreated with caffeine (1–10–100 µM) for 24 h and then co-treated with LPS (150 ng/ml) for 2 h. Caffeine downregulated TNF-α **(A)**, IL-6, **(B)** and IL-1β **(C)** mRNA levels, up-regulated by the LPS challenge. Caffeine 1 and 100 µM increased BDNF mRNA levels, reduced by LPS **(D)**. Values were reported as mean ± SD; *n* = 4. Data were analyzed by one-way ANOVA and the Tukey *post hoc* test for multiple comparisons. *p < 0.05 *vs.* control; †*p* < 0.05 *vs.* LPS 150 ng/ml; ^‡^ *p* < 0.05 *vs.* LPS + caffeine 1 μM; **p < 0.05 *vs.* LPS + caffeine 10 µM.

**FIGURE 3:**
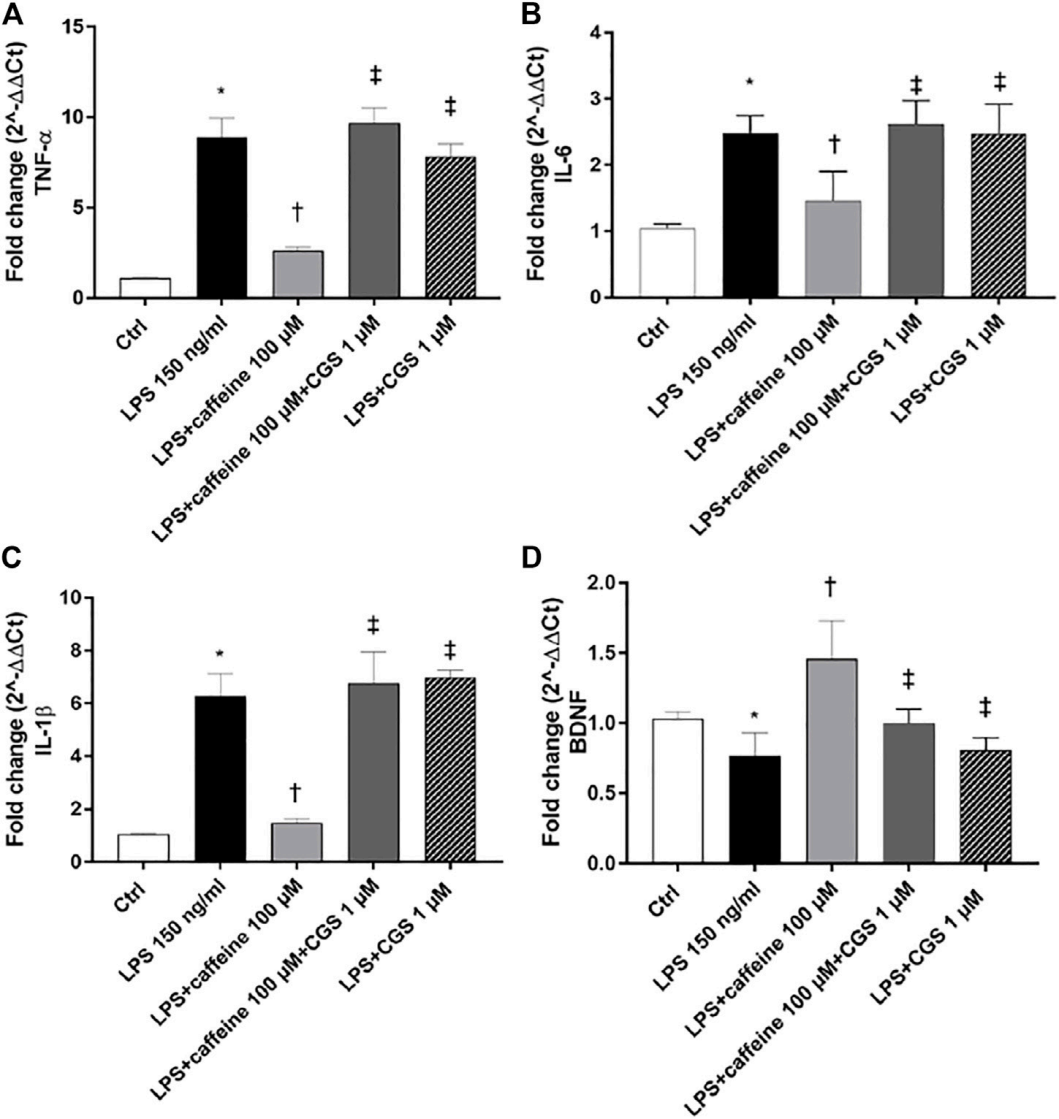
The selective A_2A_ agonist CGS 21680 counteracts the effects of caffeine. Cells were pretreated with caffeine (100 µM) and/or CGS (1 µM) for 24 h and then co-treated with LPS (150 ng/ml) for 2 h. The treatment with CGS (LPS + CGS; LPS + caffeine + CGS) did not counteract LPS-mediated effects as regard as inflammatory cytokines **(A–C)** or BDNF **(D)**. Contrarily, pretreatment with caffeine (LPS + caffeine) reduced inflammatory cytokines mRNA expression and increased BDNF mRNA levels. Values are reported as mean ± SD; *n* = 4. Data were analyzed by one-way ANOVA and the Tukey *post hoc* test for multiple comparisons.*p < 0.05 *vs.* control; ^†^ *p* < 0.05 *vs.* LPS 150 ng/ml; ^‡^ *p* < 0.05 *vs.* LPS + caffeine 100 µM.

Treatment with LPS (150 ng/ml) led to a significant (*p* < 0.05) increase of TNF-α, IL-6, and IL-1β mRNA levels, compared to control cells. Caffeine, at all tested concentrations, significantly (*p* < 0.05) reduced the expression of these inflammatory cytokines, in comparison with LPS-treated cells (Figure 2A–C). Moreover, LPS treatment significantly (*p* < 0.05) reduced the BDNF expression in ARPE-19 cells, compared to untreated cells. This effect was significantly (*p* < 0.05) counteracted by caffeine as demonstrated by the BDNF mRNA expression in ARPE-19 cells, damaged by LPS (Figure 2D). We found that caffeine upregulated the BDNF expression in ARPE-19 cells exposed to LPS, even though the intermediate concentration (10 µM) did not have effect (Figure 2D). Furthermore, as shown in Figure 3 (A, B, and C), the A_2A_ selective receptor agonist, CGS 21680 (1 µM), led to a significant (*p* < 0.05) increase in mRNA levels of inflammatory cytokines, compared to cells treated with caffeine 100 µM. Moreover, while caffeine (100 µM) was able to restore significantly (*p* < 0.05) the BDNF mRNA levels, the A_2A_ selective receptor agonist CGS (1 µM) counteracted the effect of caffeine on the BDNF expression (Figure 3D).

### Effects of Caffeine on p-NFκB p65 Nuclear Translocation

**FIGURE 4:**
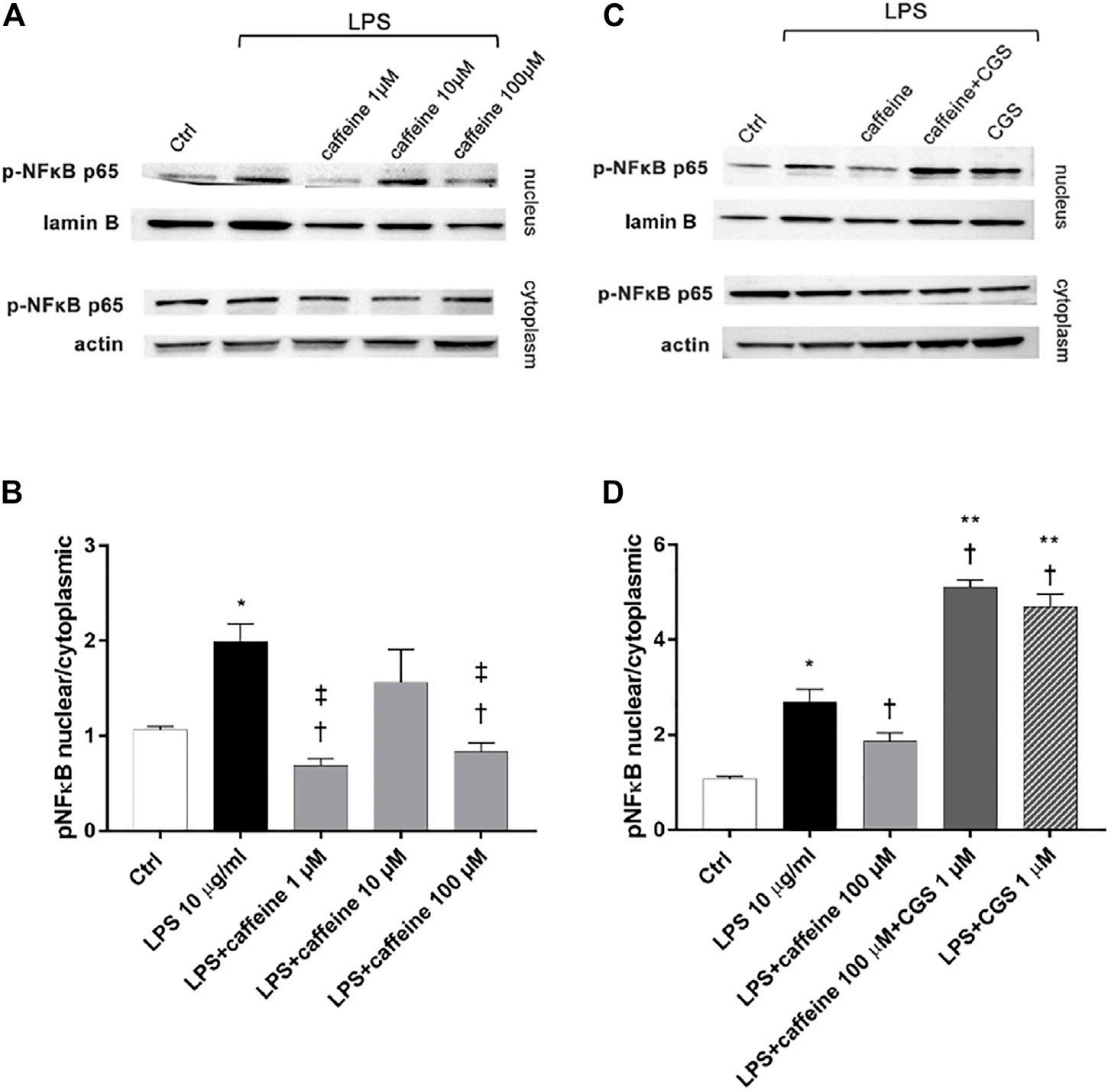
Caffeine reduces nuclear translocation of p-NFκB elicited by LPS. ARPE-19 were pretreated with caffeine (1–10–100 µM) and/or CGS (1 µM) for 24 h and then co-treated with LPS (10 μg/ml) for 2 h **(A–C)** Representative blots of nuclear and cytoplasmic proteins **(B–D)** Densitometry of the p-NFκB p65 nuclear translocation in treated cells (ratio of nuclear p-NFκB p65/lamin B and cytoplasmic p-NFκB p65/actin). Each bar represents the mean value ±SD; *n* = 4. Data were analyzed by one-way ANOVA and the Tukey *post hoc* test for multiple comparisons. *p < 0.05 *vs.* control; †*p* < 0.05 *vs.* LPS 10 μg/ml; ‡*p* < 0.05 *vs.* LPS + caffeine 10 μM, **p < 0.05 vs LPS + caffeine 100 µM.

After 2 h, LPS (10 μg/ml) exposure significantly (*p* < 0.05) increased the nuclear translocation of p-NFκB p65, compared to control. Pretreatment for 24 h with caffeine (1 and 100 µM) significantly (*p* < 0.05) reduced the nuclear translocation of p-NFκB p65, confirming the anti-inflammatory effect of this compound in retinal pigment epithelial cells, challenged with LPS. However, caffeine 10 µM did not counteract the activation of NFκB (Figure 4A,B). As shown in Figure 4 (C and D), the selective A_2A_ agonist CGS (1 µM) counteracted the anti-inflammatory effects of caffeine on ARPE-19 cells damaged by LPS, as regards as p-NFκB p65 nuclear translocation.

### Effects of Caffeine on BRB Integrity

**FIGURE 5:**
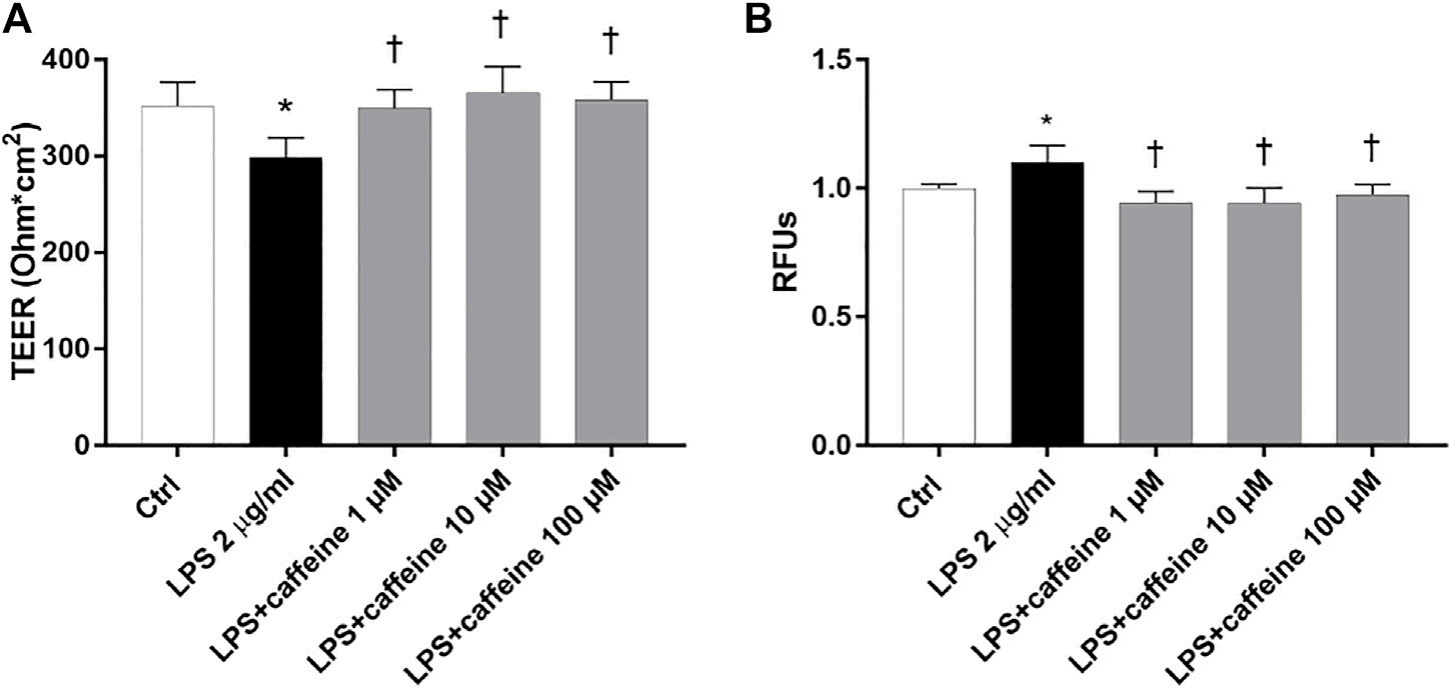
Caffeine protects the ARPE-19 cell monolayer from LPS-induced damage. ARPE-19 were pretreated with caffeine (1–10–100 µM) for 24 h and co-treated with LPS (2 µ/ml) for 24 h **(A)** Caffeine, at all concentrations, increased TEER values, which were reduced by LPS **(B)** Measurement of apical-to-basolateral Na-F permeability. Representative Na-F permeability measured after 15 min. Values are reported as mean ± SD; *n* = 4. Data were analyzed by one-way ANOVA and the Tukey *post hoc* test for multiple comparisons. *p < 0.05 *vs.* control; †*p* < 0.05 *vs.* LPS 2 μg/ml; ^‡^ *p* < 0.05 *vs.* LPS + caffeine 100 µM.

To investigate the effect of caffeine on BRB integrity, we assessed the transepithelial electrical resistance (TEER) and immunostaining of ZO-1 tight junction, in ARPE-19. After 24 h, the LPS challenge (2 μg/ml) significantly (*p* < 0.05) decreased TEER values, in comparison to untreated cells (control) (Figure 5A). Caffeine, at all tested concentrations, significantly (*p* < 0.05) increased TEER values, in comparison to LPS-treated cells, meaning a restored BRB integrity (Figure 5A). These data were also confirmed by measurement of the apical-to-basolateral permeability of sodium fluorescein (Na-F). Treatment with caffeine (1–100 µM), at all considered time points (5′-15′ and 30'; 5′ and 30′ Supplementary Figure S1, Supplementary Material), led to a significant (*p* < 0.05) reduction of cell permeability, significantly (*p* < 0.05) increased by the LPS challenge (Figure 5B).

**FIGURE 6:**
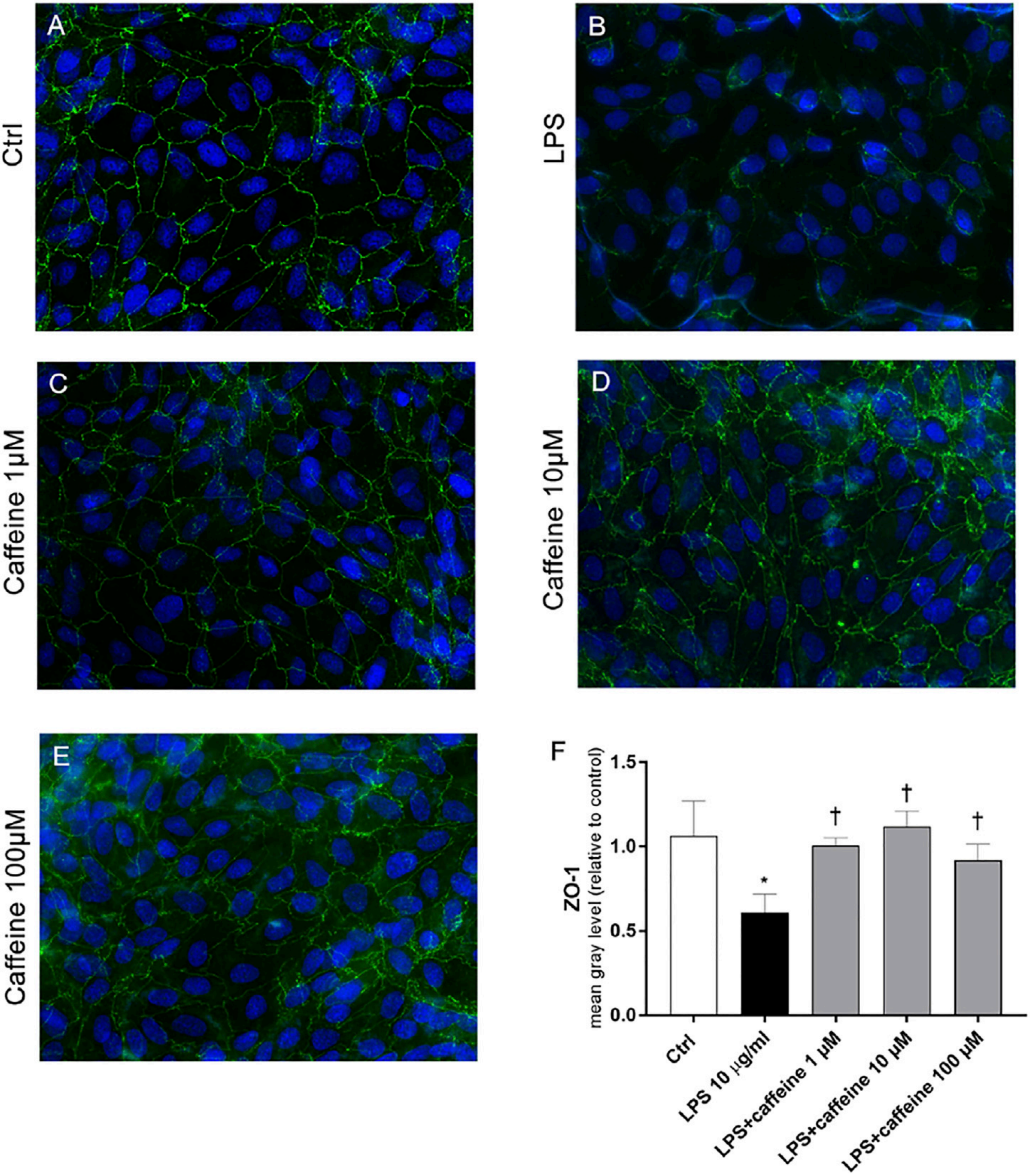
Caffeine re-establishes the BRB integrity through modulation of ZO-1. ARPE-19 were pretreated with caffeine (1–10–100 µM) for 24 h and subsequently co-treated with LPS (10 μg/ml) for 72 h. Caffeine, at all concentrations, increased the expression of ZO-1 protein, which was significantly reduced by LPS **(A–E)** Representative images for the ZO-1 expression in ARPE-19 after treatment with LPS and caffeine. ZO-1 was labeled with FITC (green); nuclei were labeled with DAPI (blue). Images were acquired at 20× magnification **(F)** Fluorescence semi-quantification of ZO-1 protein (mean gray levels). Values are reported as mean ± SD; *n* = 4. Data were analyzed by one-way ANOVA and the Tukey *post hoc* test for multiple comparisons. *p < 0.05 *vs.* control; ^†^ *p* < 0.05 *vs.* LPS 10 μg/ml.

According to the instrumental and spectroscopic evaluation of ARPE-19 monolayer integrity, after 72 h, LPS (10 μg/ml) significantly (*p* < 0.05) decreased the ZO-1 expression (Figure 6B), compared to control cells (Figure 6A). The treatment with caffeine (1, 10, 100 µM) reverted this LPS-related damage, reestablishing the ZO-1 expression and BRB integrity (Figure 6C–E).

### Effects of Caffeine in Retinal I/R-Injured Mice

**FIGURE 7:**
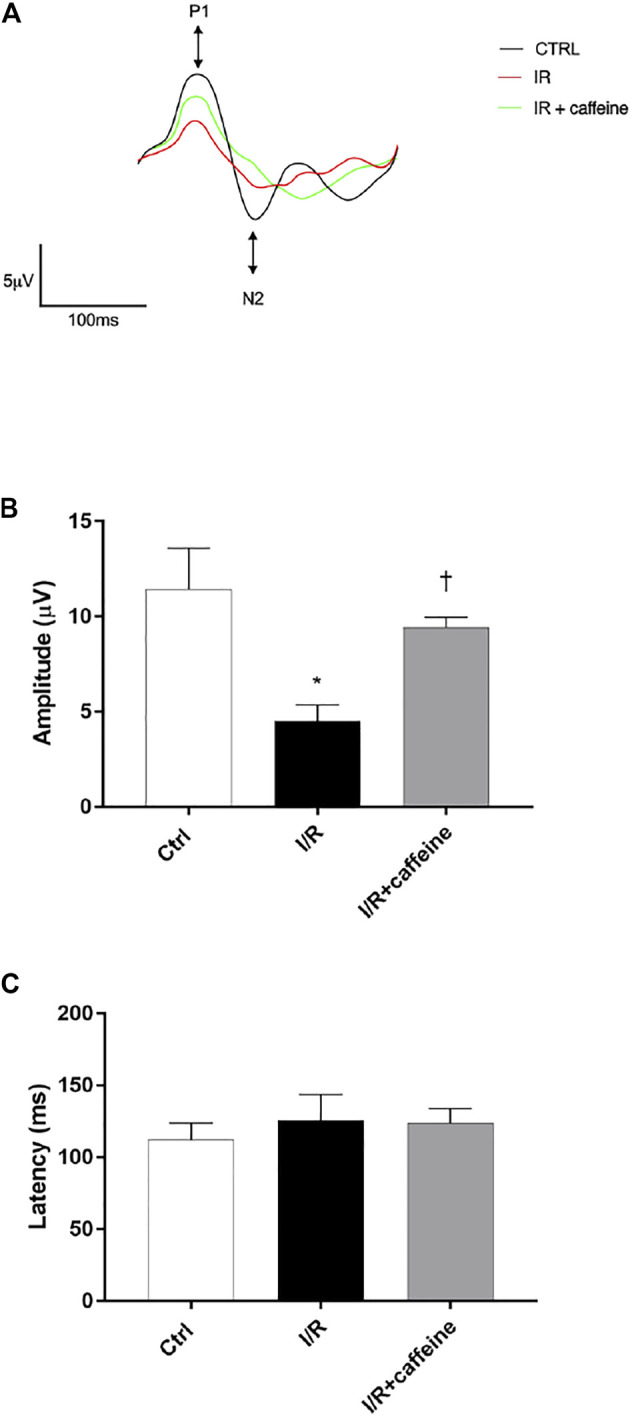
Caffeine counteracts RGC loss of function induced by I/R injury in mouse retinas. RGC function was assessed by the pattern electroretinogram (PERG). **(A)** Representative waveforms of PERG of all experimental groups (C57BL/6J mouse control (Ctrl), I/R, and I/R + caffeine). Each waveform, deriving from PERG recordings, was analyzed for the peak-to-trough amplitude (P1-N2). **(B)** Comparison between PERG amplitude values (µV) and latency values (ms). **(C)** of control, I/R, and I/R + caffeine. In each panel, bars represent the mean values ±S.D; *n* = 4. One-way ANOVA analysis was performed followed by the Tukey *post hoc* test. *p < 0.05 *vs*. Ctrl; ^†^ *p* < 0.05 *vs*. I/R.

**FIGURE 8:**
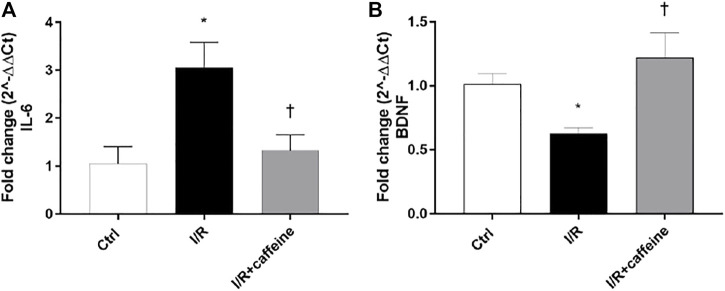
Caffeine counteracts inflammation and elicits the BDNF expression in I/R mice. After 72 h, caffeine was able to revert the upregulation of IL-6 mRNA levels **(A)** elicited by I/R damage in mouse retinas. BDNF mRNA levels were upregulated by caffeine, compared to I/R mice **(B)**. Values were reported as mean ± SD;*n* = 4. Data were analyzed by one-way ANOVA and the Tukey *post hoc* test for multiple comparisons. *p < 0.05 *vs.* control; ^†^ *p* < 0.05 *vs.* I/R.

We analyzed the effect of caffeine on the RGC function in I/R mice after 72 h, by means of PERG measurements (Figure 7 A, representative retinal waveforms). As expected, the PERG amplitude decreased (∼50%) in I/R-injured mice, in comparison to control mice, while caffeine-treated mice showed a PERG amplitude significantly (*p* < 0.05) higher than I/R-injured mice (Figure 7B). Indeed, the average value of the PERG amplitude was 11.41 μV in the control group, in agreement with previous studies (Porciatti et al., 2010; Romano et al., 2020), while the average value of the PERG amplitude of I/R mice was significantly (*p* < 0.05) reduced to 4.51 μV, compared to the control retina. It is noteworthy that the average value of the PERG amplitude of I/R caffeine-treated mice was 9.41 μV, suggesting a protective effect of caffeine in terms of RGC function (Figure 7B). No significant changes were observed in PERG latency in all experimental groups, as expected, considering the short time after the injury (Figure 7C). As shown in Figure 8A, I/R injury elicited significant (*p* < 0.05) increase of the IL-6 mRNA expression, that was significantly (*p* < 0.05) counteracted by caffeine treatment. Furthermore, I/R damage significantly (*p* < 0.05) downregulated the mRNA expression of BDNF in mouse retinas, while caffeine significantly induced (*p* < 0.05) the BDNF mRNA expression compared to the I/R group (Figure 8B).

## Discussion

Caffeine is approved for clinical use, and it is indicated for the treatment of the apnea of prematurity (Abdel-Hady, 2015). Increasing interest on caffeine has risen from several *in vitro* and *in vivo* evidence of neuroprotective effects in the model of brain neurodegenerative diseases (Chen et al., 2001, Chen et al., 2008a; Singhal et al., 2015; Akomolafe et al., 2017). Moreover, some clinical trials are focusing on the therapeutic potential of this natural stimulatory compound in Alzheimer’s disease and Parkinson’s disease (NCT01190735; NCT05009199; NCT01190735; NCT04570085). Previous studies reported the anti-inflammatory, anti-oxidative, and neuroprotective properties of caffeine (Jung et al., 2017; Kolahdouzan and Hamadeh, 2017; Metro et al., 2017). Only few studies have investigated the effects of caffeine on retinal diseases. On this regards, the Coimbra Eye Study, an epidemiological cross-sectional study, evidenced an inverse correlation between consumption of caffeine and AMD progression. The authors concluded that caffeine could be a promising nutritional supplement for slow-down of the AMD progression (Campochiaro et al., 2016; Raimundo et al., 2018), highlighting the need for further pre-clinical pharmacological studies on caffeine and retinal diseases. Based on this evidence, we evaluated, for the first time, the anti-inflammatory and neuroprotective effect of caffeine, in human RPE cells and mouse retinas challenged with LPS (Ozal et al., 2018) and ischemia/reperfusion (Conti et al., 2021), respectively.

LPS elicits retinal inflammation through activation of TLR-4, which is expressed in RPE cells, leading to inflammatory cytokine release and causing several degenerative processes (Kumar et al., 2004; Chen et al., 2017; Klettner et al., 2020). It has been demonstrated that caffeine suppresses the LPS-induced inflammatory response, reducing the expression of several inflammatory mediators in different types of cells, such as microglia and monocyte/macrophage-like cells (Kang et al., 2012; Hwang et al., 2016). In accordance with these findings, we demonstrated that caffeine exerted anti-inflammatory and neuroprotective effects also in RPE cells and in the retina of mice after LPS and ischemia insults, respectively. Caffeine reduced the mRNA expression of TNF-α, IL-6, and IL-1β in RPE cells after LPS exposure. Furthermore, caffeine significantly counteracted the p-NFκB p65 nuclear translocation in RPE cells exposed to LPS, showing a biphasic effect, already observed in other systems (Su et al., 2013) and with other compounds (Calabrese, 2016; Kurano et al., 2016). Finally, caffeine protected RPE cells also through the upregulation of BDNF, as already reported in different systems (Costa et al., 2008; Sallaberry et al., 2013; Lao-Peregrín et al., 2017). Moreover, retinal BDNF was also upregulated by caffeine in our retinal I/R *in vivo* model; in this paradigm, we also demonstrated that caffeine reduced IL-6 mRNA levels in comparison to I/R mice. BDNF is strongly reduced in several neurodegenerative processes both in the brain (Howells et al., 2000; Leyhe et al., 2008) and in the retina (Johnson et al., 2009; Kimura et al., 2016; Oddone et al., 2017; Platania et al., 2019; Conti et al., 2021). The overexpression of BDNF, and the reduced expression of IL-6, elicited by caffeine treatment in the retinal I/R model, could explain the protection of RGCs showed by PERG analysis in mice. Moreover, several studies demonstrated that some retinal diseases such as AMD are characterized by the abnormal expression and irregular distribution of tight junction proteins in RPE cells (Du et al., 2013). Hence, it is well known that LPS affects the epithelial integrity (Zheng et al., 2018; He et al., 2019), reducing the expression of ZO-1 protein and diminishing TEER values, in ARPE-19 cell monolayers (Chen et al., 2017; Zou et al., 2018; Hernandez et al., 2021). In this study, we confirmed that the LPS insult reduced the ARPE-19 monolayer integrity, as shown by instrumental (TEER measurements), spectroscopic (NaF permeability assays), and immunocytochemistry analyses. The pretreatment with caffeine brought TEER values and NaF permeability to levels shown by ARPE-19-negative control cells. Furthermore, caffeine restored the ZO-1 expression, in ARPE-19 exposed to LPS treatment. The present findings are in line with the previous studies, which demonstrated that caffeine is able to prevent cell–cell interaction network disruption, not only as regard as the retinal barrier but also in the blood–brain barrier (BBB) (Chen et al., 2008a, Chen et al., 2008b; Maugeri et al., 2017). Caffeine has an interesting pharmacological profile, and several studies demonstrated that the antagonism of A_2A_ receptors modulates neuroinflammation in retinal ganglion cells (Madeira et al., 2016; Boia et al., 2017), in microglia (Madeira et al., 2018), and in neuronal cells (Rebola et al., 2011). Based on this evidence, we supported the hypothesis that caffeine exerts its neuroprotective and anti-inflammatory effects through A_2A_ receptor signaling because the agonist (CGS 21680) counteracted the effects of caffeine in RPE cells exposed to LPS. In conclusion, we demonstrated that caffeine was able to protect RPE cells and RGCs from damage elicited by LPS and ischemia, respectively, showing a key role of BDNF. These findings suggest that caffeine may be a potential candidate for retinal degeneration treatment.

## Data Availability

The original contributions presented in the study are included in the article/Supplementary Material; further inquiries can be directed to the corresponding author.
