# Supplementary material for: Caffeine Protects Against Retinal Inflammation
Source: Front Pharmacol. 2022 Jan 6;12:824885. doi: 10.3389/fphar.2021.824885 (PMC8773454; doi:10.3389/fphar.2021.824885)
Supplement: Supplementary file 1 [file DataSheet1.docx]

**SUPPLEMENTARY MATERIAL**

**Caffeine protects against retinal inflammation**

Federica Conti^1†^, Francesca Lazzara^1†^, Giovanni Luca Romano^1,2^, Chiara Bianca Maria Platania^1^, Filippo Drago^1,2^ and Claudio Bucolo^1,2*^

^1^Department of Biomedical and Biotechnological Sciences, School of Medicine, University of Catania, Catania, Italy; ^2^Center for Research in Ocular Pharmacology-CERFO, University of Catania, Catania, Italy

† These authors have contributed equally to this work


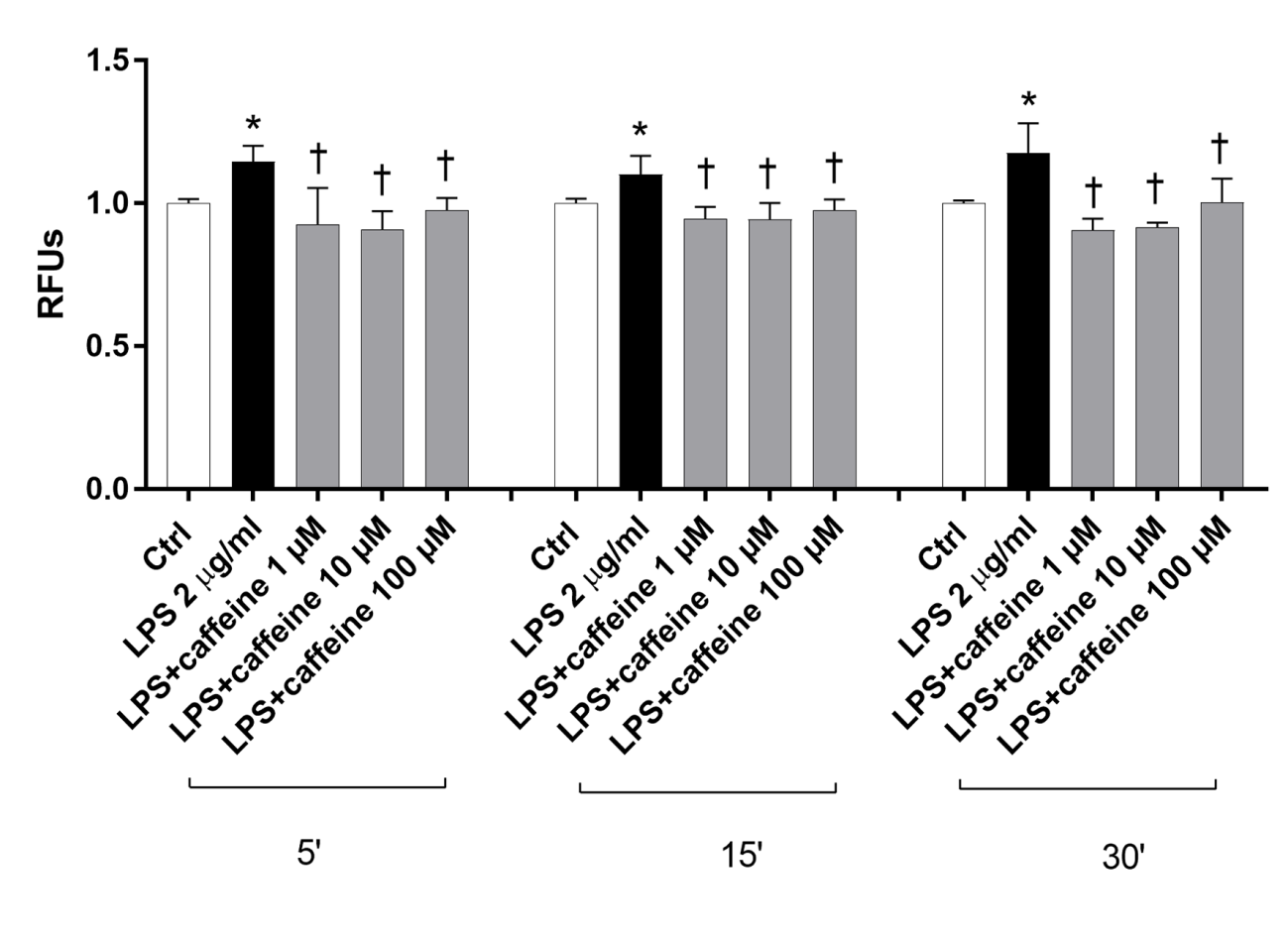


**Figure 1S:** Measurement of apical-to-basolateral Na-F permeability. Na-F permeability was measured after 5, 15, and 30 min through fluorescence measurement. At all time points, caffeine (1-100 µM) was able to reduce permeability, increased by LPS. Values are reported as mean ± SD; n=4. Data were analyzed by one-way ANOVA and Tukey post-hoc test for multiple comparisons. *p<0.05 *vs.* control; † p < 0.05 *vs.* LPS 2 µg/ml; ‡ p<0.05 *vs.* LPS+caffeine 100 µM.
